# Online Learning Approach for Predictive Real-Time Energy Trading in Cloud-RANs

**DOI:** 10.3390/s21072308

**Published:** 2021-03-25

**Authors:** Wan Nur Suryani Firuz Wan Ariffin, Xinruo Zhang, Mohammad Reza Nakhai, Hasliza A. Rahim, R. Badlishah Ahmad

**Affiliations:** 1 Faculty of Electronic Engineering Technology, Universiti Malaysia Perlis, Arau 02600, Malaysia; badli@unimap.edu.my; 2 School of Computer Science and Electronic Engineering, University of Essex, Wivenhoe Park, Colchester CO4 3SQ, UK; xinruo.zhang@essex.ac.uk; 3 Department of Informatics, Centre for Telecommunications Research, King’s College London, Aldwych WC2B 4BG, UK; reza.nakhai@kcl.ac.uk; 4 Advanced Communication Engineering, Centre of Excellence (ACE), Universiti Malaysia Perlis, Kangar 01000, Malaysia; 5 Advanced Computing, Centre of Excellence (AdComp), Universiti Malaysia Perlis, Arau 02600, Malaysia

**Keywords:** cloud radio access network, combinatorial multi-armed bandit, online learning, energy trading

## Abstract

Constantly changing electricity demand has made variability and uncertainty inherent characteristics of both electric generation and cellular communication systems. This paper develops an online learning algorithm as a prescheduling mechanism to manage the variability and uncertainty to maintain cost-aware and reliable operation in cloud radio access networks (Cloud-RANs). The proposed algorithm employs a combinatorial multi-armed bandit model and minimizes the long-term energy cost at remote radio heads. The algorithm preschedules a set of cost-efficient energy packages to be purchased from an ancillary energy market for the future time slots by learning both from cooperative energy trading at previous time slots and by exploring new energy scheduling strategies at the current time slot. The simulation results confirm a significant performance gain of the proposed scheme in controlling the available power budgets and minimizing the overall energy cost compared with recently proposed approaches for real-time energy resources and energy trading in Cloud-RANs.

## 1. Introduction

Denser site deployment has been contemplated as a key enabling technology that can support the mushrooming of mobile data traffic and meet the demands of high-data-rate communications for next-generation wireless communication networks [1]. In contrast, conventional base stations (BSs) consume 80% of the electricity [2], as, in the BSs, all the radio and baseband processing functions are coordinated in the second-generation (2G) radio access network (RAN) architecture. Subsequently, the radio and baseband processing functions are divided into two separate nodes, i.e., remote radio head (RRH) and baseband processing unit (BBU), in the development of the third-generation (3G) and fourth-generation (4G) distributed radio access network (Distributed-RAN) architecture. Nevertheless, Distributed-RAN is incompetent in dealing with tremendous growths in data traffic to deliver high-bandwidth, low-latency, and cost-efficient services [3], and is incapable of supporting the demands of the quality of expectation (QoE) and quality of service (QoS) [4] for the fifth-generation (5G) of mobile communication systems. Cloud radio access networks (Cloud-RANs) have been regarded as a promising solution, owing to their superiority in reducing the capital expenditure (CAPEX) and operational expenditure (OPEX) of the network operators with the centralization and cloudification of BBUs and their corresponding RRHs. Cloud-RANs can solve the limitations of the Distributed-RAN architecture in terms of expanding the network scalability, simplifying network management and maintenance, optimizing network performance, reducing energy consumption, and enhancing spectrum efficiency [3]. In a Cloud-RAN architecture, the conventional BSs are physically detached into two parts: BBUs, which are grouped as a cloud processing unit (CU) for designing all coordination and energy trading strategies, and the remaining RRHs, which are in charge of all radio frequency (RF) operations [5]. Even beamforming is designed in the CU; RRHs consume an enormous portion of electricity to amplify and transmit RF signals to users in order to satisfy their data-rate and energy requirements. However, due to the large number of densely deployed RRHs, with each serving a time-varying number of users in a highly dynamic wireless environment, the amount of energy demanded by the wireless network operators from the energy generation (EG) plants will be highly variable and statistically unknown over different times of the day. Equipping the RRHs with green energy technology that harvests energy from natural sources, such as wind and sunlight, to power next-generation mobile communication networks can significantly contribute to the reduction of the global carbon footprint [6]. However, the uncertain nature of renewable energy supply coupled with dynamic user energy demand necessitates the integration of green energy supply with the conventional grid to maximally benefit the network operator [7,8,9,10,11,12,13,14,15]. These random variations in electricity demand increase the OPEX of the energy generation process because the EG plants must maintain an instantaneous balance between the aggregate demand for electricity and the total power generated as a whole [7]. Hence, the operators need to routinely control the operation of the wireless network based on the well-known operating characteristics of the conventional EG plants. Deviation from the operating points of the EG plants to provide compensating variations in order to maintain the balance increases the total OPEX of the EG plants, which will, in turn, reflect on the OPEX of the network operators.

The operational time frame of the grid can be generally divided into regulation, load following, and unit commitment. During each one of these time frames, suitable reserved energy sources are dispatched to correct the imbalance between the generation and the demand. The EG sources reserved for load following, which are deployed on a slower time scale than the regulating frames, are used to accommodate for causes of variability and uncertainty, e.g., due to traffic energy demand and renewable energy generation, during the regular operation of the grid. Although the ramping and energy needed to follow the variations and uncertainties can be supplied by the ancillary energy markets, the insufficient ramping capability of the base low-cast conventional power plants can significantly inflate the price of the energy dispatched by expensive peaking EG units with fast ramp rates. Using conventional regulation units to compensate for uncertain abrupt ramps in energy demand is among the most expensive services. Hence, efficient control mechanisms are required to be developed for the flexibility in the EG fleet in order to maintain their cost-aware reliable operation under variability and uncertainty.

This paper focuses on designing an intelligent control mechanism for the steep ramps in energy demand in wireless cellular networks to minimize the long-term energy cost. We introduce an online learning approach for price-aware energy procurement at RRHs by supplying the load-following EG reserves within advance energy trading offers based on possible forthcoming variations and uncertainties in the energy demand. As the energy demand varies from low to peak values during different hours, the proposed strategy is designed to avoid paying for high peak-time energy cost by purchasing energy in advance at a lower off-peak price to reduce the OPEX. The proposed approach anticipates the future energy demand (surplus) at each RRH and prepares for purchasing (selling) the energy from the hour-ahead/day-ahead market (to the grid) before the actual demand (surplus) emerges. In this way, the EG units will have more time to regulate their electricity generation process according to the demand with slower ramp rates and, consequently, at lower prices.

### 1.1. Related Works

The authors in [7] first investigated the energy efficiency problem in a coordinated multipoint (CoMP) system powered by a smart gird. They formed the problem formulation for the proposed system as a simplified two-level Stackelberg game and concluded that such a design significantly reduces the OPEX. Equipping the end-user with renewable energy devices and accounting for the varying electricity price, the authors in [8] developed an energy trading algorithm to maximally benefit the network operator while satisfying the energy demand of end-users in a grid/renewable energy hybrid network. To take advantage of two-way energy trading with the grid and cooperative transmission, the authors in [9] proposed an aggregator-aided joint communication and energy cooperation strategy in the CoMP networks powered by both grid and renewable energy. In [10], the authors designed a joint real-time energy trading and cooperative transmission mechanism based on convex optimization techniques in a smart-grid-powered CoMP system. In [11], the authors studied energy trading in a more general setting, including trading among a set of storage units and the grid from the perspective of noncooperative game theory, and they proposed an algorithm that achieves at least one Nash equilibrium point. By assuming the availability of varying hourly profiles of the energy demand of base stations and renewable generation as well as the day-ahead knowledge of hourly varying electricity prices, the authors of [12] minimized the electricity bill in cellular base stations powered jointly by a smart grid and locally harvested solar energy. The authors of [13] integrated the CoMP system with a simultaneous wireless information and power transfer (SWIPT) concept and proposed a joint energy trading and partial cooperation design based on sparse beamforming, accounting for limited-capacity backhaul links in a green Cloud-RAN by minimizing the instantaneous energy cost without integrating reinforcement learning. The authors of [14] investigated the optimal power flow problem for smart micro-grids in a distributed manner and adopted an alternating direction method of multipliers to ensure the global optimum of the semidefinite programming (SDP) problem. It can be perceived that an abstract idea of the combinatorial multi-armed bandit (CMAB) approach was firstly tackled in [15] by introducing two iterative energy trading algorithms to search for a set of cost-efficient energy packages in ascending and descending order of package sizes and assuming invariability of wireless channel circumstances. Consequently, the study in [16] proposed a CMAB approach for energy trading in the cellular network to support the unpredictable wireless channel conditions to further lessen the total energy cost over a finite time horizon.

### 1.2. Main Contributions

This paper’s main contributions to real-time energy resource and energy trading in Cloud-RAN environments are summarized as follows:

- A joint energy trading and clustering technique to account for limited-capacity backhaul links in a green Cloud-RAN with a SWIPT system was proposed in [13]. However, their design was based on myopic optimization of semidefinite programming (SDP) (i.e., minimizing the instantaneous energy cost for the current time only) without any learning process for future demand provisioning. Furthermore, their proposed design cannot cope with the time-varying system dynamics, since they considered no temporal dynamic of the energy demand and cost over time and provided no solution for the look-ahead energy purchase decisions.
- In contrast to [15], this paper develops a combinatorial upper confidence bound (CUCB) algorithm as a prescheduling mechanism to maintain cost-aware reliable operation in CRANs to handle the variability and uncertainty of both the electrical generation and the intrinsic characteristics of the cellular communication system. This paper predicts the best possible combination of energy packages to be purchased for the next time slot by exploring the rewards of new combinations of energy packages within given trials at the current time slot and exploiting the past captured information on rewards of super arms from the previous time slots to optimize long-term averaged rewards.
- Differently from the system model proposed in [16], this paper considers a downlink Cloud-RAN with SWIPT, where the RRHs concurrently transfer satisfied data beams to information users and requested energy beams to active energy users. Furthermore, this paper also integrates a sparse beamforming technique to iteratively remove the cooperative links between the RRHs and the active information users based on the renewable power budgets and front-haul link capacity limitations at the individual RRHs. The clustering technique has been confirmed to enhance energy efficiency and decrease the total energy cost of the RRH in pragmatic Cloud-RANs [17]. In contrast to their CMAB approach, this paper estimates the imminent energy demands by dynamically deciding on an optimal set of super arms by exploring all of the possible minimal combinatorial energy packages to be purchased from the day-ahead market, thus diminishing the risk of regret factors.

This work’s novel contribution is the development of a sequential learning algorithm that adaptively tracks the temporal variations of energy demands and makes predictive decisions on look-ahead energy purchases in dynamically changing environments with unknown statistics to asymptotically minimize the time-averaged overall energy cost in the long run. The proposed algorithm anticipates the future energy demands of the distributed RRHs in the Cloud-RAN and schedules these demands by invoking the various power plants well in advance so that higher energy prices at peak demand times are curtailed. The proposed algorithm does not require any other description of usage patterns or statistical distribution of stochastic events. It performs foresighted optimization based on online learning during the operation. It only uses the past captured data on averaged accumulated rewards for predicting the energy consumption at the next period based on the proposed strategy.

### 1.3. Organization and Notations

The rest of this paper is structured as follows. The system model for the downlink Cloud-RAN with SWIPT and the energy management model are introduced in the Section 2. In Section 3, the problem of real-time collaborative energy trading at an individual time frame is formulated and then transformed into a numerically tractable form. The predictive energy trading strategy is proposed in Section 4. Numerical simulation results are interpreted in Section 5. Finally, Section 6 summarizes the proposed work.

**Notation** **1.** *w, w, and W⪰0, respectively, denote a scalar w, a vector w, and a positive semidefinite matrix W. $C^{n\times m}$, ${\left(.\right)}^{H}$, tr(.), and E indicate the sets of n-by-m dimensional complex matrices, the complex conjugate transpose operators, the trace operators, and the expected value, respectively. ${∥.∥}_{p}$ represents the $\ell_{p}$-norm of a vector and ${∥.∥}_{0}$ denotes the number of non-zero entries in the vector. Notice that the duration of a time frame is normalized to one and the normalized energy unit, i.e., $Js^{-1}$, is assumed in this paper. Therefore, in this paper, the terms “power” and “energy” are mutually interchangeable.*

## 2. System Model

Consider a downlink transmission Cloud-RAN with SWIPT from *N* RRHs towards $K_{i}$ information users (IUs), $K_{e}$ active energy users (EUs), and $K_{e}^{\left[\mathrm{idle}\right]}$ idle EUs, respectively, over a shared bandwidth. Notice that the active EUs located within the energy-serving area of an RRH can exploit the energy-carrying signals directly from that particular RRH. In contrast, the idle EUs located outside any energy-serving area of the RRHs can only scavenge energy from the ambient radio frequency signals for self-sustainability [13]. Each RRH is equipped with *M* antennas, and the individual IUs and EUs have one single antenna. Based on perfect knowledge of channel state information (CSI), the CU coordinates all the resource management and energy trading strategies for the RRHs and administers all the IUs’ data to the corresponding RRHs finite-capacity front-haul links. Remark that, under the perfect CSI assumption, all the channel properties of the downlink Cloud-RAN communication links, i.e., path loss, scattering, fading, shadowing, etc., are assumed to be perfectly known at both the IU and EU terminals.

Let $L_{b}=\left\{1,\cdots ,N\right\}$, $L_{i}=\left\{1,\cdots ,K_{i}\right\}$, $L_{e}=\left\{1,\cdots ,K_{e}\right\}$, and $L_{e}^{\left[\mathrm{idle}\right]}=\left\{1,\cdots ,K_{e}^{\left[\mathrm{idle}\right]}\right\}$ denote, respectively, the set of indexes of the RRHs, the IUs, the active EUs, and the idle EUs. The amount of energy flow in this paper depends on the data-rate requirements by the IUs, the wireless energy transfer requirements by the active EUs, and the harvested energy requirements from the environment by the idle EUs, whereas the amount of data flow depends only on the IUs. Let us divide the long-term period *T* into discrete time slots, indexed as T={1,⋯,T}, and define F={1,⋯,F} and K={1,⋯,K} as the set of indexes of the frames within a time slot and the set of indexes of the learning trials within a frame, respectively. The channel is assumed to vary across frames, but remains invariant within each frame. This paper proposes an online learning algorithm that iteratively alternates between designing the overall transmission strategy using convex optimization and preparing for future energy demand from the day-/hour-ahead market via online learning, i.e., a CMAB approach, to avoid steep ramps in the energy generation plant and to minimize the long-term energy cost.

### 2.1. Energy Management Model

Similarly to [13], it is assumed that at least one renewable energy generator, i.e., solar panel or/and wind turbine, is installed in the vicinity of each RRH. In this setup, none of the RRHs are equipped with any frequently rechargeable storage devices. Furthermore, bidirectional energy trading with the primary grid is enabled at the individual RRHs. Thus, the RRHs can purchase energy in the day-/hour-ahead market during off-peak hours at a lower price and/or in the spot market during peak hours at a higher price, and the surplus energy can also be sold back to the grid at an agreed-upon price. Let $B_{n}^{\left[\mathrm{spot}\right]}$, $B_{n}^{\left[\mathrm{ahead}\right]}$, $S_{n}$, and $E_{n}$ denote, at time slot t,t∈T, the amount of real-time energy purchases from the spot-market for the *n*-th RRH to cover an instantaneous energy shortage, the amount of look-ahead energy purchases from the day-/hour-ahead market at the end of previous time slot “t − 1”, the amount of surplus energy to be traded back to the primary grid, and the amount of renewable energy generation at the *n*-th RRH, respectively. In addition, let $P_{n}^{\left[\mathrm{Tx}\right]}$ be the total transmit power and $P_{n}^{\left[\mathrm{circ}\right]}$ be the total power consumption of the hardware circuits at the *n*-th RRH. Furthermore, in any frame, the total energy consumption at the *n*-th RRH, i.e., $P_{n}^{\left[\mathrm{total}\right]}$, is constrained as

(1) $$P_{n}^{\left[\mathrm{total}\right]}=P_{n}^{\left[\mathrm{Tx}\right]}+P_{n}^{\left[\mathrm{circ}\right]}=B_{n}^{\left[\mathrm{spot}\right]}+B_{n}^{\left[\mathrm{ahead}\right]}-S_{n}+E_{n}.$$

By viewing from the perspective of supply and demand, let us assume $\pi^{\left[\mathrm{spot}\right]}\geq \pi^{\left[\mathrm{ahead}\right]}\geq \pi^{\left[\mathrm{sell}\right]}\geq \pi^{\left[\mathrm{renew}\right]}$, where $\pi^{\left[\mathrm{spot}\right]}$, $\pi^{\left[\mathrm{ahead}\right]}$, $\pi^{\left[\mathrm{sell}\right]}$, and $\pi^{\left[\mathrm{renew}\right]}$ denote the price of purchasing (selling) per unit energy of $B_{n}^{\left[\mathrm{spot}\right]}$, $B_{n}^{\left[\mathrm{ahead}\right]}$, $S_{n}$, and generating per unit energy of $E_{n}$ (by averaging the capital expenses and OPEX of renewable devices over their lifetime), respectively. Then, the cumulative energy cost procured by the *n*-th RRH at the *k*-th trial, k∈K of the frame f,f∈F at the time slot t,t∈T, i.e., $B_{n}^{\left[\mathrm{total}\right]}\left(k\right)$, is given by

(2) $$\begin{array}{ccc} B_{n}^{\left[\mathrm{total}\right]}\left(k\right) & & =\pi^{\left[\mathrm{spot}\right]}B_{n}^{\left[\mathrm{spot}\right]}\left(k\right)+\pi^{\left[\mathrm{ahead}\right]}B_{n}^{\left[\mathrm{ahead}\right]}\left(k\right)\\ & & -\pi^{\left[\mathrm{sell}\right]}S_{n}\left(k\right)+\pi^{\left[\mathrm{renew}\right]}E_{n}\left(k\right),\;\forall n\in L_{b}. \end{array}$$

### 2.2. Downlink Transmission Model

Let $w_{i}={\left[w_{1i}^{H},\cdots ,w_{Ni}^{H}\right]}^{H}\in C^{MN\times 1}$ and $v_{e}={\left[v_{1e}^{H},\cdots ,v_{Ne}^{H}\right]}^{H}\in C^{MN\times 1}$ be defined, respectively, as the set of indexes of the beamforming vector from all RRHs towards the *i*-th IU, $i\in L_{i}$ and the *e*-th active EU, $e\in L_{e}$, where $w_{ni}\in C^{M\times 1}$ and $v_{ne}\in C^{M\times 1}$ represent the beamformer from the *n*-th RRH to the *i*-th IU and the *e*-th active EU, respectively. In addition, let $h_{i}={\left[h_{1i}^{H},\cdots ,h_{Ni}^{H}\right]}^{H}\in C^{MN\times 1}$ denote the set of indexes of the channel vector between all RRHs and the *i*-th IU, where $h_{ni}\in C^{M\times 1}$ denotes the channel vector from the *n*-th RRH to the *i*-th IU. Accordingly, the signal collected at the *i*-th IU, $i\in L_{i}$, can be expressed as the summation of the dedicated information-carrying signal, the inter-user interference induced by other non-devoted information beams, the interference provoked by the energy-carrying signals assigned to all active EUs, and the additive white Gaussian noise at the *i*-th IU as 

(3) $$\begin{array}{c} y_{i}=h_{i}^{H}w_{i}s_{i}^{\left[\mathrm{IU}\right]}+\sum_{\begin{array}{c} j\neq i\\ j\in L_{i} \end{array}}h_{i}^{H}w_{j}s_{j}^{\left[\mathrm{IU}\right]}+\sum_{e\in L_{e}}h_{i}^{H}v_{e}s_{e}^{\left[\mathrm{EU}\right]}+n_{i}. \end{array}$$

Due to the fact that energy beams carry no information, only the data of IUs will be delivered via the front-haul links. Without loss of generality, $E\left(s_{i}^{\left[\mathrm{IU}\right]}\right)=E\left(s_{e}^{\left[\mathrm{EU}\right]}\right)=1$ is assumed, and the signal-to-interference-plus-noise ratio (SINR) at the *i*-th IU, $i\in L_{i}$, is formulated as

(4) $${\mathrm{SINR}}_{i}^{\left[\mathrm{IU}\right]}=\frac{|h_{i}^{H}w_{i}{|}^{2}}{\sum_{j\in L_{i},j\neq i}|h_{i}^{H}w_{j}{|}^{2}+\sum_{e\in L_{e}}{\left|h_{i}^{H}v_{e}\right|}^{2}+\sigma_{i}^{2}},$$

where $|h_{i}^{H}w_{i}{|}^{2}$ indicates the desired power received at the *i*-th IU and $|w_{i}{|}^{2}$ is the required transmit power at the RRHs. Let us define the scheduling arrangements between the *i*-th IU and the *n*-th RRH for partial cooperation [18], i.e., ${∥∥w_{ni}{∥}_{2}^{2}∥}_{0}$, as 

(5) $${∥∥w_{ni}{∥}_{2}^{2}∥}_{0}=\left\{\begin{array}{c} 0,\;\mathrm{if}\;∥w_{ni}{∥}_{2}^{2}=0,\\ 1,\;\mathrm{if}\;∥w_{ni}{∥}_{2}^{2}\neq 0, \end{array}\right.$$

where $∥w_{ni}{∥}_{2}^{2}=0$ betokens that the *i*-th IU is not selected to be supported by the *n*-th RRH and, hence, the front-haul link between the CU and the *n*-th RRH is not employed for joint data transmission to the *i*-th IU. Hence, the front-haul link capacity consumption of the *n*-th RRH is expressed as

(6) $$C_{n}^{\left[\mathrm{front}\right]}=\sum_{i\in L_{i}}{∥∥w_{ni}{∥}_{2}^{2}∥}_{0}R_{i},\;\forall n\in L_{b},$$

where $R_{i}=\log_{2}\left(1+{\mathrm{SINR}}_{i}^{\left[\mathrm{IU}\right]}\right)$ is the achievable data-flow rate (bit/s/Hz) for the *i*-th IU and directly depends on the transmit power and the wireless channel fading condition. The total energy received by the *e*-th active EU, $e\in L_{e}$, is defined as

(7) $$G_{e}^{\left[\mathrm{EU}\right]}=\eta \left(|g_{e}^{H}v_{e}{|}^{2}+\sum_{j\in L_{e},j\neq e}|g_{e}^{H}v_{j}{|}^{2}+\sum_{i\in L_{i}}{\left|g_{e}^{H}w_{i}\right|}^{2}\right),$$

where the terms on the right-hand side of (7) represent the intended energy-carrying signal for the *e*-th active EU, the inter-user interference caused by all other non-desired energy beams, and the inter-user interference caused by information beams, respectively. Let 0≤η≤1 denote the conversion efficiency to convert the harvested RF energy into the functional electrical energy form, and $g_{e}={\left[g_{1e}^{H},\cdots ,g_{Ne}^{H}\right]}^{H}\in C^{MN\times 1}$ indicates the set of indexes of the channel vector between all the RRHs and the *e*-th active EU. The collective energy that can be harvested from the ambiances and atmospheres by the *z*-th idle EU, $z\in L_{e}^{\left[\mathrm{idle}\right]}$, is presented as

(8) $$G_{z}^{\left[\mathrm{ET}-\mathrm{idle}\right]}=\eta (\sum_{i\in L_{i}}|f_{z}^{H}w_{i}{|}^{2}+\sum_{e\in L_{e}}|f_{z}^{H}v_{e}{|}^{2}),$$

where $f_{z}={\left[f_{1z}^{H},\cdots ,f_{Nz}^{H}\right]}^{H}\in C^{MN\times 1}$ represents the set of indexes of the channel vector between all the RRHs and the *z*-th idle EU.

## 3. Real-Time Energy Trading on an Individual Time Frame

This paper relies on foresighted optimization based on CMAB learning to minimize the long-term average energy cost. In accordance with (2), the total energy cost at a given trial of a frame within a time slot is determined by four parameters, i.e., $B_{n}^{\left[\mathrm{spot}\right]}$, $S_{n}$, $E_{n}$, and $B_{n}^{\left[\mathrm{ahead}\right]}$. It is assumed that the amount of renewable energy supply $E_{n}$ is given at the beginning of each time slot, whereas $B_{n}^{\left[\mathrm{ahead}\right]},\forall n\in L_{b}$ is determined in advance at the end of the previous time slot via the proposed online learning algorithm, i.e., Algorithms 1 and 2 in Section 4, to prepare for future demands.

### 3.1. Problem Formulation

Let us define $P_{n}^{\left[\mathrm{Tx}\right]}=\sum_{i\in L_{i}}\left|\right|w_{ni}{\left|\right|}_{2}^{2}+\sum_{e\in L_{e}}\left|\right|v_{ne}{\left|\right|}_{2}^{2}$ as the total power transmitted by the *n*-th RRH to its scheduled users and the degree of partial cooperation among RRHs as

$$\begin{array}{ccc} P^{\left[\mathrm{coop}\right]} & & =(\sum_{i\in L_{i}}{∥∥w_{1i}{∥}_{2}^{2}∥}_{0}+\cdots +\sum_{i\in L_{i}}{∥∥w_{Ni}{∥}_{2}^{2}∥}_{0})\\ & & +(\sum_{e\in L_{e}}{∥∥v_{1e}{∥}_{2}^{2}∥}_{0}+\cdots +\sum_{e\in L_{e}}{∥∥v_{Ne}{∥}_{2}^{2}∥}_{0}). \end{array}$$

To minimize the average energy cost, let us consider the following cooperative energy trading model in each trial of a frame within a time slot for the given $B_{n}^{\left[\mathrm{ahead}\right]}$ as 

(9) $$\begin{array}{ccc} \min_{\begin{array}{c} w_{ni},v_{ne},\\ B_{n}^{\left[\mathrm{spot}\right]},S_{n} \end{array}} & & \alpha P^{\left[\mathrm{coop}\right]}+\sum_{n\in L_{b}}P_{n}^{\left[\mathrm{Tx}\right]}+\sum_{n\in L_{b}}\left\{B_{n}^{\left[\mathrm{spot}\right]}\right\}\\ s.t. & & C1:{\mathrm{SINR}}_{i}^{\left[\mathrm{IU}\right]}\geq \gamma_{i},\;\forall i\in L_{i},\\ & & C2:G_{e}^{\left[\mathrm{EU}\right]}\geq P_{e}^{\left[\min\right]},\;\forall e\in L_{e},\\ & & C3:G_{z}^{\left[\mathrm{EU}-\mathrm{idle}\right]}\geq P_{z}^{\left[\mathrm{idle}\right]}\;\forall z\in L_{e}^{\left[\mathrm{idle}\right]},\\ & & C4:P_{n}^{\left[\mathrm{Tx}\right]}\leq E_{n}+B_{n}^{\left[\mathrm{ahead}\right]}+B_{n}^{\left[\mathrm{spot}\right]}-S_{n}-P_{n}^{\left[\mathrm{circ}\right]}\\ & & C5:P_{n}^{\left[\mathrm{Tx}\right]}\leq P_{n}^{\left[\mathrm{Tmax}\right]},\;\forall n\in L_{b},\\ & & C6:C_{n}^{\left[\mathrm{front}\right]}\leq C_{n}^{\left[\mathrm{limit}\right]},\;\forall n\in L_{b},\\ & & C7:\sum_{n\in L_{b}}B_{n}^{\left[\mathrm{ahead}\right]}+\sum_{n\in L_{b}}B_{n}^{\left[\mathrm{spot}\right]}\leq P_{\mathrm{CU}}^{\left[\max\right]}-P_{\mathrm{CU}}^{\left[\mathrm{circ}\right]}\\ & & C8:B_{n}^{\left[\mathrm{spot}\right]}\geq 0,,\;\forall n\in L_{b},\\ & & C9:S_{n}\geq 0,\;\forall n\in L_{b}, \end{array}$$

where α≥0 is the maximal energy cost in the front-haul link for the degree of partial cooperation among RRHs. C1 guarantees the minimum SINR requirements $\gamma_{i}$ for the *i*-th IUs. $P_{e}^{\left[\min\right]}$ in C2 indicates the minimal energy demanded by the active EUs, whereas $P_{z}^{\left[\mathrm{idle}\right]}$ in C3 is the minimal conditions of energy harvested from the ambiance and atmospheres by the idle EUs. C4 betokens that the individual RRHs’ power budget restrains the total transmit power as per (1), while C5 emphasizes that the total transmit power is upper-limited by the maximum transmit power permitted, i.e., $P_{n}^{\left[\mathrm{Tmax}\right]}$, at the *n*-th RRH. C6 expresses the front-haul link capacity limitations for the individual RRHs. C7 implies the restriction for the total power provided by the grid to the RRHs, where $P_{\mathrm{CU}}^{\left[\mathrm{circ}\right]}$ is the hardware circuit power consumption and $P_{\mathrm{CU}}^{\left[\max\right]}$ is the maximum power generated by the grid at the CU [19]. C8 and C9 are the non-negative constraints set for the optimization variables.

### 3.2. Re-Weighted $\ell_{1}$-Norm and Semidefinite Programming

The optimization problem in (9) is an NP-hard (nondeterministic polynomial time) problem due to the non-convexity of the $\ell_{0}$-norm term in the objective function and the constraints C1 and C6. These non-convexity terms can be reformulated by using one of the powerful convex optimization techniques, i.e., semidefinite programming (SDP). Note that the $\ell_{1}$-norm approximation is commonly adopted in compressed sensing to handle $\ell_{0}$-norm optimization problems [20]. Then, let us consider the following property:

(10) $$x^{H}Ax=\mathrm{tr}\left(xx^{H}A\right)=\mathrm{tr}\left(Axx^{H}\right).$$

From a mathematical point of view, the property in (10) can be interpreted as the inner vector product being equal to the trace of the outer product. If A=I, then

(11) $$x^{H}x=\mathrm{tr}\left(xx^{H}\right).$$

By adding this property, denoting $H_{i}=h_{i}h_{i}^{H}$, $G_{e}=g_{e}g_{e}^{H}$, and $F_{z}=f_{z}f_{z}^{H}$, and specifying the rank-one semidefinite matrices as $W_{i}=w_{i}w_{i}^{H}$ and $v_{e}=v_{e}v_{e}^{H}$ in the optimization problem, the constraint C1 can be reformulated as

(12) $$\begin{array}{ccc} & & C1:\frac{|h_{i}^{H}w_{i}{|}^{2}}{\sum_{j\in L_{i},j\neq i}|h_{i}^{H}w_{j}{|}^{2}+\sum_{e\in L_{e}}{\left|h_{i}^{H}v_{e}\right|}^{2}+\sigma_{i}^{2}}\geq \gamma_{i},\;\;\forall i\in L_{i}, \end{array}$$

(13) $$\begin{array}{ccc} & & C1:\mathrm{tr}\left(H_{i}W_{i}\right)\geq \gamma_{i}\sum_{j\in L_{i},j\neq i}\mathrm{tr}\left(H_{i}W_{j}\right)+\gamma_{i}\sum_{e\in L_{e}}\mathrm{tr}\left(H_{i}v_{e}\right)+\gamma_{i}\sigma_{i}^{2},\;\;\forall i\in L_{i}. \end{array}$$

Following a procedure similar to that in [13], the intractability of the $\ell_{0}$-norm term in the objective function and constraint C6 is overcome by approximating by their respective re-weighted $\ell_{1}$-norms [20], as follows:

(14) $$\begin{array}{ccc} P^{\left[\mathrm{coop}\right]} & & \approx \sum_{i\in L_{i}}{∥[\xi_{1i}∥w_{1i}{∥}_{2}^{2}]∥}_{1}+\cdots +\sum_{i\in L_{i}}{∥[\xi_{Ni}∥w_{Ni}{∥}_{2}^{2}]∥}_{1}\\ & & +\sum_{e\in L_{e}}{∥[\kappa_{1e}∥v_{1e}{∥}_{2}^{2}]∥}_{1}+\cdots +\sum_{e\in L_{e}}{∥[\kappa_{Ne}∥v_{Ne}{∥}_{2}^{2}]∥}_{1}\\ & & =\sum_{n\in L_{b}}\left(\sum_{i\in L_{i}}\xi_{ni}\mathrm{tr}\left(w_{i}w_{i}^{H}D_{n}\right)+\sum_{e\in L_{e}}\kappa_{ne}\mathrm{tr}\left(v_{e}v_{e}^{H}D_{n}\right)\right), \end{array}$$

(15) $$\begin{array}{c} C_{n}^{\left[\mathrm{front}\right]}\approx \sum_{i\in L_{i}}{∥[\xi_{ni}∥w_{ni}{∥}_{2}^{2}]∥}_{1}R_{i}=\sum_{i\in L_{i}}\xi_{ni}\mathrm{tr}\left(w_{i}w_{i}^{H}D_{n}\right)R_{i}, \end{array}$$

where $D_{n}≜\mathrm{diag}\left(\overset{(n-1)M}{\overset{︷}{0,...,0,}}\overset{M}{\overset{︷}{1,...,1,}}\overset{(N-n)M}{\overset{︷}{0,...,0}}\right)⪰0,\forall n\in L_{b}$, is used for extracting the corresponding beamformer $w_{ni}$. $\xi_{ni}$ and $\kappa_{ne}$, respectively, are the weighting factors associated with the *n*-th RRH and the *i*-th IU/the *e*-th active EU, which will be updated as per the re-weighted $\ell_{1}$-norm method algorithm in [13] to iteratively remove the collaborative links between the RRHs and the IUs/active EUs in the circumstances of front-haul link capacity limitations at the individual RRHs. Hence, the non-convex problem formulation introduced in (9) can be modified to a convex optimization problem with significantly reduced complexity [21] after relaxing the rank-one constraints of $\mathrm{rank}(W_{i})=1$ and $\mathrm{rank}(v_{e})\leq 1$, as (16).

**Lemma** **1.** *The optimal solutions to the problems* (16) satisfy rank $(W_{i}^{*})=1$ and rank $(v_{e}^{*})\leq 1$ with a probability of one.

**Proof.**  The proof is straightforward by following similar steps to those in [13].    □

As per [22], the interior point methods for solving SDP have polynomial (quadratic) worst-case complexity and are superb for medium- and large-scale problems, e.g., those bounded by O(logn), where *n* is the problem size. Furthermore, as the size of the optimization problem grows large, the computational complexity tends to grow more slowly and even remains almost constant according to [23]. Hence, with increasing size of the problem, e.g., an increasing total number of RRHs, users, and per-RRH antennas, the number of iterations needed to solve the optimization problem grows sub-linearly with the size of the problem, and even tends to remain almost constant.

(16) $$\begin{array}{ccc} \min_{\begin{array}{c} W_{i},\\ V_{e},S_{n},\\ B_{n}^{\left[\mathrm{spot}\right]} \end{array}} & & \sum_{n\in L_{b}}\left(\sum_{i\in L_{i}}\xi_{ni}\mathrm{tr}\left(W_{i}D_{n}\right)+\sum_{e\in L_{e}}\kappa_{ne}\mathrm{tr}\left(V_{e}D_{n}\right)\right)\\ & & +\left(\sum_{i\in L_{i}}\mathrm{tr}\left(W_{i}\right)+\sum_{e\in L_{e}}\mathrm{tr}\left(V_{e}\right)\right)+\sum_{n\in L_{b}}\left\{B_{n}^{\left[\mathrm{spot}\right]}\right\}\\ s.t. & & C1:\mathrm{tr}\left(H_{i}W_{i}\right)\geq \gamma_{i}\sum_{j\in L_{i},j\neq i}\mathrm{tr}\left(H_{i}W_{j}\right)+\\ & & \;\;\gamma_{i}\sum_{e\in L_{e}}\mathrm{tr}\left(H_{i}V_{e}\right)+\gamma_{i}\sigma_{i}^{2},\;\forall i\in L_{i},\\ & & C2:\mathrm{tr}\left(G_{e}V_{e}\right)+\sum_{\begin{array}{c} j\in L_{e},\\ j\neq e \end{array}}\mathrm{tr}\left(G_{e}V_{j}\right)+\sum_{i\in L_{i}}\mathrm{tr}\left(G_{e}W_{i}\right)\\ & & \;\;\;\;\;\;\geq P_{e}^{\left[\min\right]}\eta^{-1},\;\forall e\in L_{e},\\ & & C3:\sum_{i\in L_{i}}\mathrm{tr}\left(F_{z}W_{i}\right)+\sum_{e\in L_{e}}\mathrm{tr}\left(F_{z}V_{e}\right)\geq P_{z}^{\left[\mathrm{idle}\right]}\eta^{-1},\\ & & \;\;\;\;\;\;\;\;\forall z\in L_{e}^{\left[\mathrm{idle}\right]},\\ & & C4:\sum_{i\in L_{i}}\mathrm{tr}\left(W_{i}D_{n}\right)+\sum_{e\in L_{e}}\mathrm{tr}\left(V_{e}D_{n}\right)\leq [E_{n}-S_{n}\\ & & \;\;+B_{n}^{\left[\mathrm{ahead}\right]}+B_{n}^{\left[\mathrm{spot}\right]}-P_{n}^{\left[\mathrm{circ}\right]}],\;\forall n\in L_{b},\\ & & C5:\sum_{i\in L_{i}}\mathrm{tr}\left(W_{i}D_{n}\right)+\sum_{e\in L_{e}}\mathrm{tr}\left(V_{e}D_{n}\right)\leq P_{n}^{\left[\mathrm{Tmax}\right]},\\ & & C6:\sum_{i\in L_{i}}\xi_{ni}\mathrm{tr}\left(W_{i}D_{n}\right)R_{i}\leq C_{n}^{\left[\mathrm{limit}\right]},\;\forall n\in L_{b},\\ & & C7:\sum_{n\in L_{b}}B_{n}^{\left[\mathrm{ahead}\right]}+\sum_{n\in L_{b}}B_{n}^{\left[\mathrm{spot}\right]}\leq P_{\mathrm{CU}}^{\left[\max\right]}-P_{\mathrm{CU}}^{\left[\mathrm{circ}\right]}\\ & & C8:B_{n}^{\left[\mathrm{spot}\right]}\geq 0,\;C9:S_{n}\geq 0,\;\forall n\in L_{b},\\ & & C10:W_{i}⪰0,\;\forall i\in L_{i},\;C11:V_{e}⪰0,\;\forall e\in L_{e}. \end{array}$$

## 4. Predictive Energy Trading Strategy

The multi-armed bandit (MAB) problem is expressed as a *J*-arm system, with each being associated with independent and identically distributed (i.i.d.) stochastic rewards. The objective is to maximize the accumulated profits by observing the associated reward of new arms during the exploration stage while simultaneously optimizing the decisions among a set of arms based on existing knowledge at the exploitation stage in multiple trials [24]. Let consider a combinatorial generalization of the classical MAB problem, where a super arm consisting of a set of *N* base arms, N⊂J, is played, and the rewards of its relevant base arms are observed individually in each trial [25].

**Figure 1:**
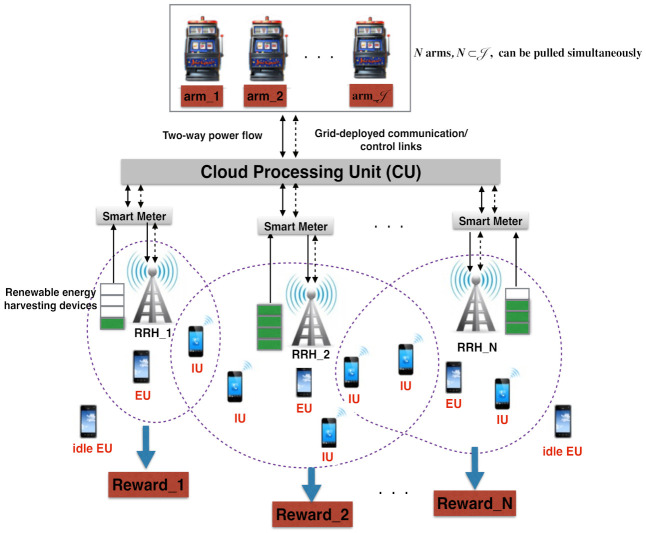
Combinatorial multi-armed bandit (CMAB) problem for predictive real-time energy trading in a cloud radio access network (Cloud-RAN) with the sparse beamforming technique.

As illustrated in Figure 1, the problem scrutinized in this paper is categorized as a combinatorial MAB problem, where a super arm is composed of *N* base arms and each base arm corresponds to an energy package purchased for an RRH from the day-/hour-ahead market at each trial k,k∈K, before the real-time energy demand. The CU adapts its cooperative energy trading strategies to the intermittent environment in the Cloud-RAN by dynamically forming super arms to maximize the averaged rewards accumulated over the period *T*, which is equivalent to lessening the averaged energy expense in the long run. Let J={1,⋯,J} be defined as a set of indexes for possible energy packages offered in the day-/hour-ahead market by the grid, and let $E^{\left[\mathrm{total}\right]}=\left\{E^{1},\cdots ,E^{J}\right\}$ denote all energy packages offered by the grid in the day-/hour-ahead market, where $E^{p}=E^{p-1}+\Delta E,\;p\in J$. Furthermore, let $A_{k}^{\left[\mathrm{set}\right]}=\left\{B_{1}^{\left[\mathrm{ahead}\right]}\left(k\right),\cdots ,B_{N}^{\left[\mathrm{ahead}\right]}\left(k\right)\right\}$ represent a super arm, i.e., a set of *N* energy packages purchased in advance for *N* RRHs from the day-/hour-ahead market, at the *k*-th trial. Let us further define the reward for the individual arms at the *n*-th RRH and the reward for the super arm at the *k*-th trial as $R(B_{n}^{\left[\mathrm{ahead}\right]}\left(k\right))$ and $R(A_{k}^{\left[\mathrm{set}\right]})$, respectively, as 

(17) $$\begin{array}{ccc} R(B_{n}^{\left[\mathrm{ahead}\right]}\left(k\right))= & & B_{n}^{\left[\mathrm{total}\right]}\left(1\right)-B_{n}^{\left[\mathrm{total}\right]}\left(k\right), \end{array}$$

(18) $$\begin{array}{ccc} R(A_{k}^{\left[\mathrm{set}\right]})= & & \sum_{n\in L_{b}}R\left(B_{n}^{\left[\mathrm{ahead}\right]}\left(k\right)\right), \end{array}$$

where $B_{n}^{\left[\mathrm{total}\right]}\left(1\right)$ and $B_{n}^{\left[\mathrm{total}\right]}\left(k\right)$ in (17) are the total energy cost incurred by the *n*-th RRH at the initial trial and the *k*-th trial of a frame, respectively. Furthermore, let $\mu_{n}^{\left[k,f,t\right]}=\left(\mu_{n,1}^{\left[k,f,t\right]},\mu_{n,2}^{\left[k,f,t\right]},\cdots ,\mu_{n,J}^{\left[k,f,t\right]}\right)$ be defined as the reward vector for the *n*-th RRH, where $\mu_{n,p}^{\left[k,f,t\right]}=R\left(B_{n}^{\left[\mathrm{ahead}\right]}\left(k\right)\right),p\in J$, is the reward associated to the *p*-th energy package in the *k*-th trial of the *f*-th frame at the *t*-th time slot.

**Figure 2:**
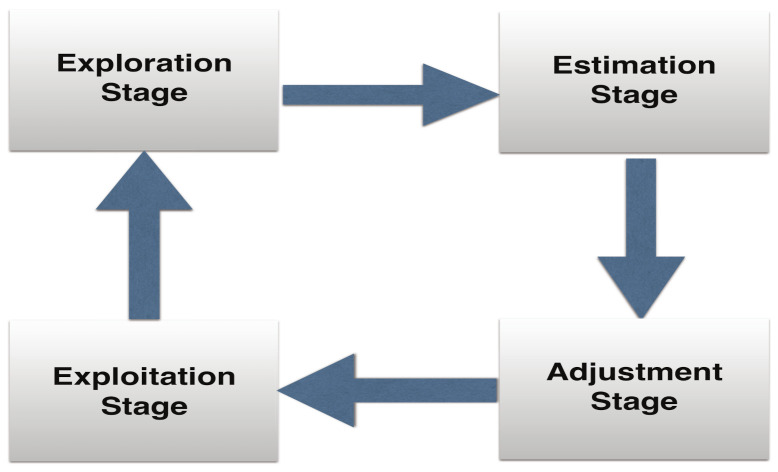
Proposed predictive energy trading strategy in a Cloud-RAN.

In the following, we propose CUCB-based [25] predictive energy trading strategy, which is shown in Figure 2 and detailed in Algorithms 1 and 2, to find the best possible combination of energy packages to be purchased from the day-/hour-ahead market for *N* RRHs for the next time slot by exploring the rewards of new combinations of energy packages within a limited number of trials at the current time slot and exploiting the past captured information on rewards of super arms from the previous time slots so that the long-term averaged rewards, i.e., the total energy cost in the long run, can be optimized.
**Algorithm 1** Super Arm Exploration1:**Initialize**: Total number of trials *K*2:**for k=1:K**3:   **Solve problem (16) for a given $B_{n}^{\left[\mathrm{ahead}\right]}\left(k\right)$,****4:** **   CU calculates $B_{n}^{\left[\mathrm{total}\right]}\left(k\right)$ as per (2), $R(B_{n}^{\left[\mathrm{ahead}\right]}\left(k\right))$ as per (17), and $R(A_{k}^{\left[\mathrm{set}\right]})$ as per (18).****5:** **      If k=1****6:** **            then                   $B_{n}^{\left[\mathrm{ahead}\right]}\left(k+1\right)=B_{n}^{\left[\mathrm{ahead}\right]}\left(k\right)+\Delta E,\;\;n\in L_{b}$.****7:** **      else if the super arm reward of all the RRHs                   $R\left(A_{k}^{\left[set\right]}\right)\leq R\left(A_{k-1}^{\left[set\right]}\right)$,****8:** **            then                   $B_{n}^{\left[\mathrm{ahead}\right]}\left(k+1\right)=B_{n}^{\left[\mathrm{ahead}\right]}\left(k-1\right)$, $\;\forall n\in L_{b}$,****9:** **      else if the individual reward for the *n*-th RRH, n∈N                   $R\left(B_{n}^{\left[\mathrm{ahead}\right]}\left(k\right)\right)\geq R\left(B_{n}^{\left[\mathrm{ahead}\right]}\left(k-1\right)\right)$                                     and                   $B_{n}^{\left[\mathrm{ahead}\right]}\left(k\right)\neq E^{J}$,****10:** **            then    $B_{n}^{\left[\mathrm{ahead}\right]}\left(k+1\right)=B_{n}^{\left[\mathrm{ahead}\right]}\left(k\right)+\Delta E$,****11:** **      else                   $B_{n}^{\left[\mathrm{ahead}\right]}\left(k+1\right)=B_{n}^{\left[\mathrm{ahead}\right]}\left(k\right)$.****12:** **      end If****13:** **   Calculate the total energy cost of all the RRHs, $\beta^{\left[k,f,t\right]}$ as                   $\beta^{\left[k,f,t\right]}=\sum_{n\in L_{b}}B_{n}^{\left[\mathrm{total}\right]}\left(k\right)$.****14:** **   Calculate the energy package index *p* at all RRHs from                   $p=\frac{B_{n}^{\left[\mathrm{ahead}\right]}\left(k\right)}{\Delta E},\;\;n\in L_{b}$.****15:** **   Update                   $\mu_{n,p}^{\left[k,f,t\right]}=R\left(B_{n}^{\left[\mathrm{ahead}\right]}\left(k\right)\right),\;\forall p\in J,\;\;n\in L_{b}$;****16:** **   Update                   $A_{k+1}^{\left[\mathrm{set}\right]}=\left\{B_{1}^{\left[\mathrm{ahead}\right]}\left(k+1\right),\cdots ,B_{N}^{\left[\mathrm{ahead}\right]}\left(k+1\right)\right\}$;****17:** **end for****18:** **      Estimated mean reward for K trials                                     ${\hat{\mu }}_{n,p}^{\left[f,t\right]}=\frac{\sum_{k=1}^{K}\mu_{n,p}^{\left[k,f,t\right]}}{K},\;\forall p\in J,n\in L_{b}$.****Algorithm 2** Main Online Learning Algorithm1:**Initialize**: Time slot count: t=0;**2:** **while t≠T do****3:** **Increment the iteration index t=t+1;****4:** **      for f=1:F****5:** **            if t=1 (initial time slot)****6:** **                  then Initialize the super arm for the first trial (k=1) as                   $A_{1}^{\left[\mathrm{set}\right]}=\left\{0_{1},\cdots ,0_{N}\right\}$,****7:**             else                   $A_{1}^{\left[\mathrm{set}\right]}=S^{*}$,**8:** **            end if****9:** **      Exploration Stage: Run Algorithm 1****10:** **      Estimation Stage:****11:** **      Calculate the mean reward vector for the frame                   ${\hat{\mu }}_{n}^{\left[f,t\right]}=\left({\hat{\mu }}_{n,1}^{\left[f,t\right]},{\hat{\mu }}_{n,2}^{\left[f,t\right]},\cdots ,{\hat{\mu }}_{n,J}^{\left[f,t\right]}\right)$, where                   ${\hat{\mu }}_{n,p}^{\left[f,t\right]}=\frac{\sum_{k=1}^{K}\mu_{n,p}^{\left[k,f,t\right]}}{K},\;\forall p\in J,n\in L_{b}$.****12:** **      Adjustment Stage:****13:** **            if $\Psi_{p}$ (number of times the *p*-th arm is played) ≠0****14:** **                  then                   adjust ${\bar{\mu }}_{n,p}^{\left[f,t\right]}={\hat{\mu }}_{n,p}^{\left[f,t\right]}+\sqrt{\frac{3\ln K}{2\Psi_{p}}}$,****15:** **            else                   ${\bar{\mu }}_{n,p}^{\left[f,t\right]}={\hat{\mu }}_{n,p}^{\left[f,t\right]},\;\;\forall p\in J,n\in L_{b}$.****16:** **            end if****17:** **      end for****18:** **Average adjusted mean reward vector over all frames                   ${\bar{\mu }}_{n}^{\left[t\right]}=\left(\frac{\sum_{f\in F}{\bar{\mu }}_{n,1}^{\left[f,t\right]}}{F},\frac{\sum_{f\in F}{\bar{\mu }}_{n,2}^{\left[f,t\right]}}{F},\cdots ,\frac{\sum_{f\in F}{\bar{\mu }}_{n,J}^{\left[f,t\right]}}{F}\right),\;\;n\in L_{b}$.****19:** **Exploitation Stage:****20:** **Average ${\bar{\mu }}_{n}^{\left[t\right]}$ over accumulated number of time slots, as                    ${\bar{\mu }}_{n}=\frac{\sum_{t^{\prime }=1}^{t}{\bar{\mu }}_{n}^{\left[t^{\prime }\right]}}{t}=\left[{\bar{\mu }}_{n,1},{\bar{\mu }}_{n,2},\cdots ,{\bar{\mu }}_{n,J}\right],\;\;n\in L_{b}$.****21:** For the next time slot: find *N* optimum arm indexes as                   $p_{n}^{*}=\underset{p}{\mathrm{argmax}}\left({\bar{\mu }}_{n,p}\right),p\in J,\;\;\forall n\in L_{b}$, and the updated super arm as                   $S^{*}=\Delta E\left[p_{1}^{*},p_{2}^{*},\cdots ,p_{N}^{*}\right]$.**22:** **end while**

Let ${\hat{\mu }}_{n}^{\left[f,t\right]}=\left({\hat{\mu }}_{n,1}^{\left[f,t\right]},{\hat{\mu }}_{n,2}^{\left[f,t\right]},\cdots ,{\hat{\mu }}_{n,J}^{\left[f,t\right]}\right)$ and ${\bar{\mu }}_{n}^{\left[f,t\right]}=\left({\bar{\mu }}_{n,1}^{\left[f,t\right]},{\bar{\mu }}_{n,2}^{\left[f,t\right]},\cdots ,{\bar{\mu }}_{n,J}^{\left[f,t\right]}\right),\forall n\in L_{b},f\in F,t\in T$ denote the estimated mean reward vector and the adjusted reward vector of individual energy packages, respectively. In the exploration stage within each frame, Algorithm 1 explores new combinations of energy packages (super arms) for the next trial based on the rewards obtained at the current and the previous trials. Once a given number of *K* trials are completed, the mean rewards for individual energy packages, i.e., ${\hat{\mu }}_{n}^{\left[f,t\right]}$, in each frame are estimated. The estimated mean rewards are, first, adjusted and averaged over a total number of *F* frames of a time slot as per step 18, then averaged again over the total number of past time slots as per step 20 [26], and, finally, used to update the super arm $S^{*}$, i.e., the optimal set of energy packages purchased from the day-ahead market, to be exploited in the next time slot, as detailed in Algorithm 2.

The proposed learning-based algorithm can be considered as a mixed online learning and convex optimization problem with linear matrix inequality constraints. The optimization problem is solved once per learning trial. Therefore, the complexity of the resulting algorithm is mainly due to the number of iterations required for solving a convex optimization problem that has polynomial worst-case complexity [22] and whose total number of learning trials depends on the dynamic range of variations in the environment.

## 5. Simulation Results

A downlink Cloud-RAN consisting of three adjacent RRHs with SWIPT towards six single-antenna IUs and six single-antenna EUs was considered in this paper. The proposed Cloud-RAN operated under the channel bandwidth of 20 MHz. All of the RRHs were installed with eight antennas and placed 500 m away from each other. The performance of the proposed scheme was assessed with K=10 trials per frame, F=10 frames per time slot, T=60 time slots, and a total number of J=20 energy packages with ΔE=100 mW, i.e., $E_{t}^{\left[\mathrm{total}\right]}=\left\{100,200,\cdots ,2000\right\}$ mW. The renewable energy generation values at the individual RRHs were $E_{1}=1.5$, $E_{2}=0.2$, and $E_{3}=0.05$ W, respectively, at a price of $\pi^{\left[\mathrm{renew}\right]}=0.02$ GBP/W. It was assumed that $\pi^{\left[\mathrm{ahead}\right]}=0.07$, $\pi^{\left[\mathrm{spot}\right]}=0.15$, and $\pi^{\left[\mathrm{sell}\right]}=0.05$ GBP/W. A correlated channel model, $h_{ni}=R^{1/2}h_{w}$, was adopted [17,27], where $h_{w}\in C^{M\times 1}$ are zero-mean circularly symmetric complex Gaussian random variables with unit variance, $R\in C^{M\times M}$ is the spatial covariance matrix, and its (m,n)-th element is given by $G_{a}L_{p}\sigma_{F}^{2}e^{-0.5\frac{{\left(\sigma_{s}\ln10\right)}^{2}}{100}}e^{j\frac{2\pi \delta }{\lambda }\left[(n-m)sin\theta \right]}e^{-2{\left[\frac{\pi \delta \sigma }{\lambda }\left(n-m\right)cos\theta \right]}^{2}},$ where $G_{a}=15$ dBi denotes the antenna gain, $L_{p}\left(\mathrm{dB}\right)=125.2+36.3\log_{10}\left(d\right)$ represents the path loss model over a distance of *d* km, $\sigma_{F}^{2}$ is the variance of the complex Gaussian fading coefficient, $\sigma_{s}=8$ dB is the log-normal shadowing standard deviation, $\sigma =2^{\circ }$ is the angular offset standard deviation, and θ is the estimated angle of departure. The simulation parameters were assumed, unless otherwise stated, to be $P_{\mathrm{CU}}^{\left[\mathrm{circ}\right]}=40$ dBm, $P_{\mathrm{CU}}^{\left[\max\right]}=50$ dBm, $P_{n}^{\left[\mathrm{circ}\right]}=30$ dBm, $P_{n}^{\left[\mathrm{Tmax}\right]}=46$ dBm, $C_{n}^{\left[\mathrm{limit}\right]}=30$ bits/s/Hz, $P_{e}^{\left[\min\right]}=-60$ dBm, $P_{z}^{\left[\mathrm{idle}\right]}=-90$ dBm [18], and η=0.5, respectively. The simulation results were accomplished via CVX [28] using an Intel i7-3770 CPU at 3.4GHz with 8 GB RAM, and the running time for each learning trial was approximately seven seconds without use of parallelization. Our proposed online learning strategy was compared against a baseline design that had no ahead-of-time energy preparation and the non-learning based design in [13], which always assumes that a fixed set of energy packages is prepared from the day-/hour-ahead market, i.e., $A^{\left[\mathrm{set}\right]}=\left\{B_{1}^{\left[\mathrm{ahead}\right]}=B_{2}^{\left[\mathrm{ahead}\right]}=B_{3}^{\left[\mathrm{ahead}\right]}\right\}=700$ mW. For fair comparison, identical constraints were applied to all the strategies.

Note that the convergence speed of the proposed online learning strategy to achieve its steady-state is based on the total number of learning trials, which also depends on the dynamic range of variations in the environment. Due to the limitations of our simulation tool, we downsized the total number of learning trials and the other simulation parameters according to the scale of our problem size. In a practical scenario, with a large number of users, the resulting amount of look-ahead energy purchased from the day-/hour-ahead market will be increased proportionally, which may increase the number of arms or increase the difference between two adjacent arms, and may also increase the number of learning trials needed to speed up the convergence. Therefore, the practical enlarged scenario does not affect the scalability of the proposed algorithm, as it may only increase the computational burden.

**Figure 3:**
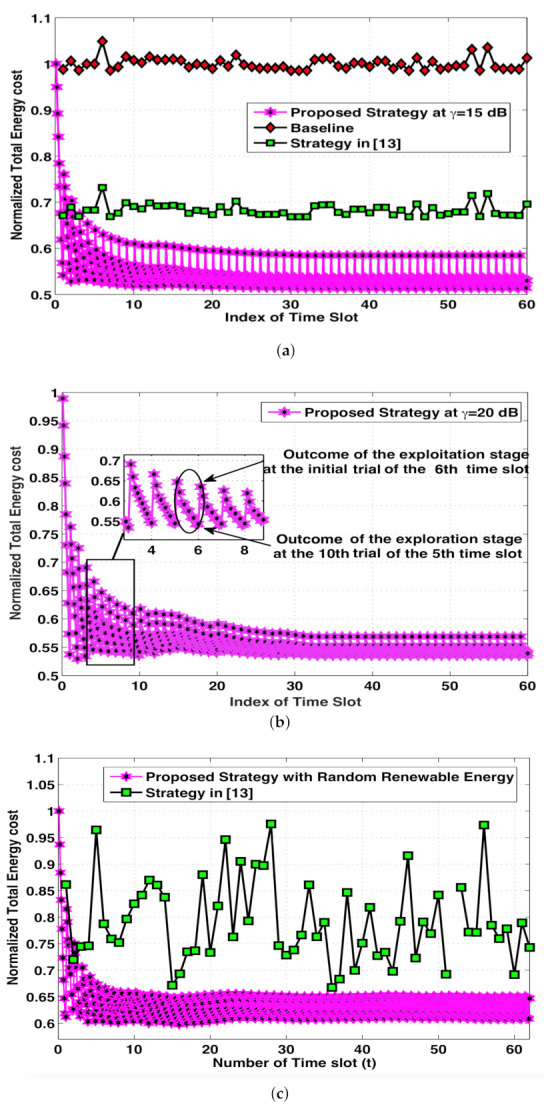
Normalized total energy cost at (**a**) γ=15, (**b**) γ=20, and (**c**) γ=20 dB with random variations in renewable generation.

Figure 3a compares the normalized total energy cost over discrete time slots for different strategies at γ=15 dB. It can be observed that, at its steady-state, the proposed strategy achieves performance gains of 43 percent and 11 percent, respectively, as compared with the baseline scheme and the design in [13], since their designs provide no adaption to the dynamic wireless channel conditions in Cloud-RANs. Figure 3b shows the normalized total energy cost of our proposed strategy at γ=20 dB. One may observe that the performance of the proposed strategy slightly degrades with increasing target SINR, i.e., from γ=15 dB to γ=20 dB. Figure 3c represents the normalized total energy cost of our proposed strategy at γ=20 dB in a more complex scenario, where it is assumed that the number of per-RRH antennas is six and the renewable energy generation at individual RRHs ranges from [0.5 2.5], [0.3 1.5], and [0.1 1.0] W, respectively.

It is clear from the Figure 3c that the performance of the proposed strategy was slightly degraded compared to Figure 3b, which was simulated in a simpler scenario. However, as the time-slot index increases, the performance of our proposed strategy indicates considerable smaller variations in total energy cost and much better average performance compared to that of [13] under the same system setup. This validates the ability of our proposed algorithm to adapt to more realistic wireless networks.

**Figure 4:**
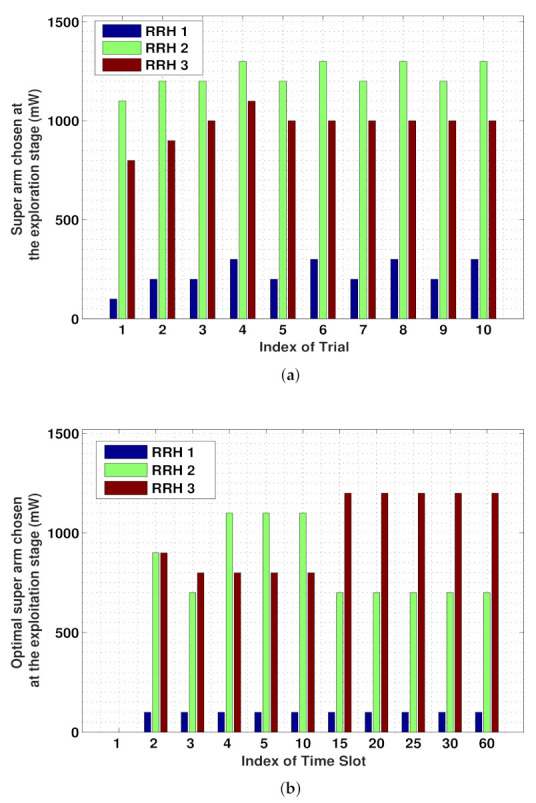
Illustration of super arm decisions according to the proposed strategy: (**a**) super arms chosen in the individual trials at the fifth time slot; (**b**) look-ahead energy purchase decisions (i.e., final super arm) for individual time slots.

Figure 4 presents in detail the procedure of a super arm being selected in accordance with Algorithms 1 and 2. Figure 4a illustrates the procedure of a super arm, i.e., an optimal set of energy packages purchased for a set of RRHs from the day-/hour-ahead market, in different trials at the fifth time slot. In each trial, a new combination of energy packages is explored on the basis of the individual and the averaged accumulated rewards obtained from the current and the previous trials, as per Algorithm 1. Figure 4b demonstrates the optimal super arm that was selected at the *t*-th time slot to be exploited as the starting point at the (t+1)-th time slot, as per Algorithm 2. It can be observed that from the 15th time slot onwards, nearly identical super arms that associate with the highest rewards for the RRHs are selected, which demonstrates the convergence of the proposed algorithm for the given simulation.

**Figure 5:**
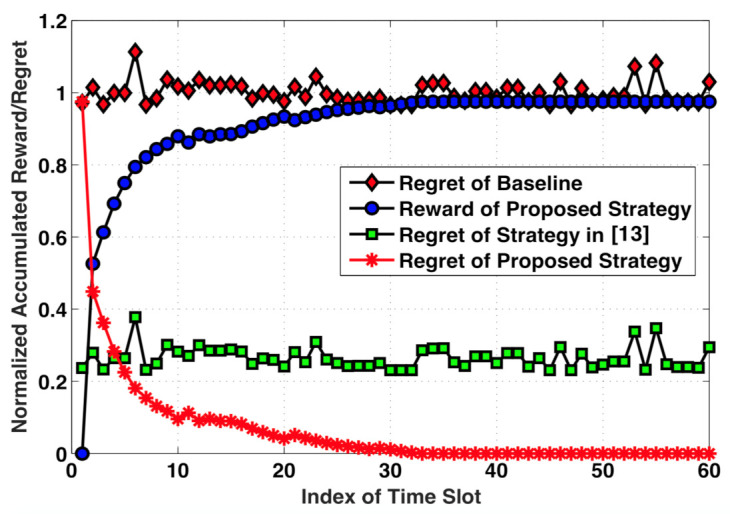
Normalized accumulated reward/regret for different strategies.

The normalized accumulated reward and regret at each time slot for different strategies are shown in Figure 5. The normalized accumulated reward at time slot *t*, denoted by $R_{t}^{\left[\mathrm{acc}\right]}$, is calculated by averaging the difference of the total energy cost at the *t*-th time slot and the initial time slot over all frames, i.e., $R_{t}^{\left[\mathrm{acc}\right]}=\frac{\sum_{f\in F}\left(\beta^{\left[k,f,1\right]}-\beta^{\left[k,f,t\right]}\right)}{F}$. In contrast, the regret of the strategies is defined as the difference in the accumulated reward between always playing the optimal super arm and playing the super arm according to the proposed strategy at the *t*-th time slot, i.e., $Q_{t}=R_{\mathrm{opt}}^{\left[\mathrm{acc}\right]}-R_{t}^{\left[\mathrm{acc}\right]}$, where $R_{\mathrm{opt}}^{\left[\mathrm{acc}\right]}$ is the accumulated reward after the convergence. Figure 5 confirms that a significant performance gap exists between the proposed strategy and the baseline scheme, as well as the design in [13]. One can conclude that, although the regret of the proposed strategy has the worst performance at the initial time slot, it declines rapidly with the continuous learning process until convergence due to the fact that the proposed strategy learns from the past captured behavior of cooperative energy trading and adapts to the dynamic wireless environment.

## 6. Conclusions

This paper proposes a predictive cooperative energy trading mechanism based on a CMAB model in a green Cloud-RAN with SWIPT, which adapts to the temporal variations of energy demands in a statistically unknown changing environment and improves its performance gain over time, with the objective of minimizing the time-averaged overall energy cost in the long run. The proposed strategy anticipates future energy demand and supplies the instantaneous energy demand at the current time slot with energy prepared in advance based on existing knowledge of uncertain wireless system dynamics at the previous time slots. The presented simulation results confirmed a reduction of the long-term running cost. Our proposed scheme outperforms a baseline scheme that purchases no ahead-of-time energy packages and a recently proposed non-learning-based design that assumes fixed energy purchases from the day-ahead market. 

## Data Availability

Not applicable.

## Abbreviations

The following abbreviations are used in this manuscript:

$L_{b}$	Index of the number *N* of BSs.
$L_{i}$	Index of the number $K_{i}$ of information users.
$L_{e}$	Index of the number $K_{e}$ of active energy users (EUs).
$L_{e}^{\left[\mathrm{idle}\right]}$	Index of the number $K_{e}^{\left[\mathrm{idle}\right]}$ of idle EUs.
$P_{n}^{\left[\mathrm{Tx}\right]}$	Total transmit power at the *n*-th RRH.
$P_{n}^{\left[\mathrm{circ}\right]}$	Hardware circuit power consumption at the *n*-th RRH.
$P_{\mathrm{CU}}^{\left[\mathrm{circ}\right]}$	Hardware circuit power consumption at the CU.
$P_{n}^{\left[\mathrm{Tmax}\right]}$	Maximum transmit power allowance of the *n*-th RRH.
$P_{\mathrm{CU}}^{\left[\max\right]}$	Maximum power provision by the grid at the CU.
T	Index of the number *T* of time slots.
F	Index of the number *F* of frames within a time slot.
K	Index of the number *K* of trials within a frame.
$B_{n}^{\left[\mathrm{ahead}\right]}$	Amount of energy purchased from the day-ahead
	market (Arm).
$B_{n}^{\left[\mathrm{spot}\right]}$	Amount of energy to be purchased from the spot-market.
$S_{n}$	Amount of excessive energy to be sold back to the grid.
$E_{n}$	Amount of renewable energy generation at the *n*-th RRH.
$B_{n}^{\left[\mathrm{total}\right]}\left(k\right)$	Total energy cost of the *n*-th RRH at the *k*-th trial.
$E^{\left[\mathrm{total}\right]}=\left\{E^{1},\cdots ,E^{J}\right\}$	All energy packages (arms) offered by the grid in the
	day-ahead market.
$\mu_{n,p}^{\left[k,f,t\right]}=R\left(B_{n}^{\left[\mathrm{ahead}\right]}\left(k\right)\right)$	Reward associated with arm $B_{n}^{\left[\mathrm{ahead}\right]}$ at the *k*-th trial of the
	*f*-th frame at the *t*-th time slot.
$A_{k}^{\left[\mathrm{set}\right]}=\left\{B_{1}^{\left[\mathrm{ahead}\right]}\left(k\right),\cdots ,B_{N}^{\left[\mathrm{ahead}\right]}\left(k\right)\right\}$	*N* energy packages purchased a day ahead at the *k*-th trial
	(super arm).
$R(A_{k}^{\left[\mathrm{set}\right]})$	Reward for the super arm $A_{k}^{\left[\mathrm{set}\right]}$ at the *k*-th trial.
$\mu_{n}^{\left[k,f,t\right]}=\left(\mu_{n,1}^{\left[k,f,t\right]},\mu_{n,2}^{\left[k,f,t\right]},\cdots ,\mu_{n,J}^{\left[k,f,t\right]}\right)$	Reward vector for the *n*-th RRH
${\hat{\mu }}_{n}^{\left[f,t\right]}=\left({\hat{\mu }}_{n,1}^{\left[f,t\right]},{\hat{\mu }}_{n,2}^{\left[f,t\right]},\cdots ,{\hat{\mu }}_{n,J}^{\left[f,t\right]}\right)$	Estimated mean reward vector
${\bar{\mu }}_{n}^{\left[f,t\right]}=\left({\bar{\mu }}_{n,1}^{\left[f,t\right]},{\bar{\mu }}_{n,2}^{\left[f,t\right]},\cdots ,{\bar{\mu }}_{n,J}^{\left[f,t\right]}\right)$	Adjusted reward vector of individual arms
$R_{t}^{\left[\mathrm{acc}\right]}$	Accumulated reward at time slot *t*.
$Q_{t}$	Regret at time slot *t*.
